# Occlusion veineuse rétinienne et syndrome d'hyperviscosité

**DOI:** 10.11604/pamj.2015.20.9.5098

**Published:** 2015-01-05

**Authors:** Samar Younes, Meriem Abdellaoui, Fadoua Zahir, Idriss A Benatiya, Hicham Tahri

**Affiliations:** 1 Service d'Ophtalmologie, CHU Hassan II, Fes, Maroc

**Keywords:** Occlusion veineuse rétinienne, hyperviscosité, rétinopathie de stase

## Abstract

Les occlusions veineuses rétiniennes secondaires aux syndromes d'hyperviscosité sont rares. Plusieurs cas d'occlusion de la veine centrale de la rétine [OVCR] compliquant une hémopathie ont été décrits, principalement au cours des polycythémies primitives ou secondaires, des lymphomes ou des leucémies. A travers cette observation, nous rapportons le cas d'un patient qui présente une OVCR de l’œil droit survenant dans le cadre d'un myélome multiple. La rétinopathie du syndrome d'hyperviscosité est liée au ralentissement circulatoire qui affecte de manière prépondérante le secteur veineux et donne un aspect de rétinopathie de stase bilatérale, avec dilatation et tortuosité de l'ensemble des veines rétiniennes. A un certain degré d'hyperviscosité, une occlusion veineuse véritable peut survenir. Le traitement comprend la réhydratation, phlébotomie, et plasmaphérèse.

## Introduction

Les occlusions veineuses rétiniennes (OVR) secondaires aux syndromes d'hyperviscosité sont rares. Depuis les premiers travaux de Mausolf et coll. en 1973, qui avaient induit une occlusion veineuse rétinienne expérimentale chez le singe en élevant artificiellement la viscosité sanguine, de nombreuses publications ont suivi mettant en évidence un lien fort entre les états d'hyperviscosités et la survenue ou l'aggravation des OVR [1]. La maladie de Kahler ou myélome multiple est une prolifération médullaire maligne de plasmocytes sécrétant une immunoglobuline monoclonale. L´atteinte rétinienne est rarement rapportée dans la littérature, et elle est due à un syndrome d´hyperviscosité sanguine responsable d´un syndrome vaso-occlusif compliquant la maladie [2].

## Patient et observation

**Figure 1:**
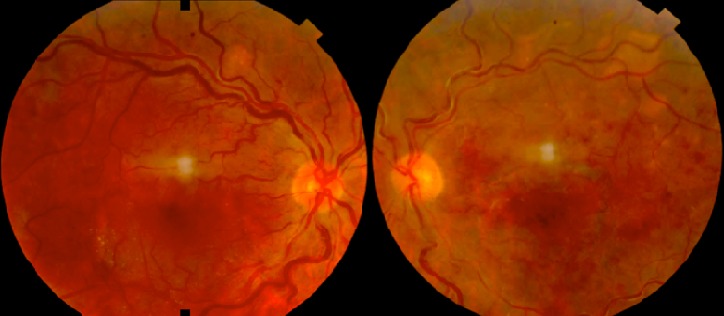
L'examen du fond d’œil révèle une rétinopathie de stase liée à l'hyperviscosité sans OVCR véritable « paraproteinaemicus fundus » de l’œil droit et une OVCR au niveau de l’œil gauche

**Figure 2:**
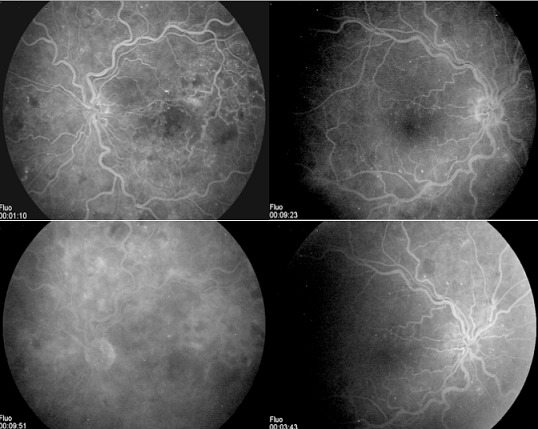
L'angiographie à la fluorescéine montre une OVCR au niveau de l’œil gauche forme œdémateuse, avec importantes dilatations et tortuosité veineuses et une rétinopathie de stase liée à l'hyperviscosité sans OVCR véritable de l’œil droit

**Figure 3:**
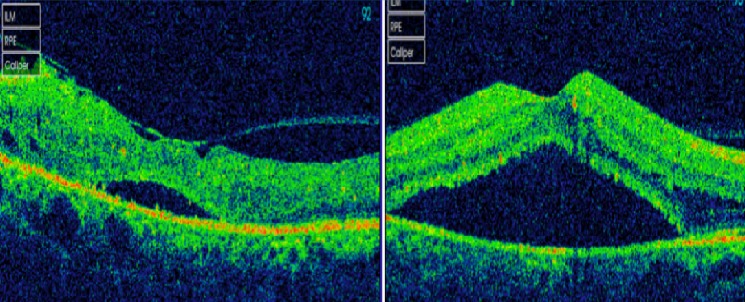
La tomographie par cohérence optique révèle la présence bilatérale du liquide sous rétinien

**Figure 4:**
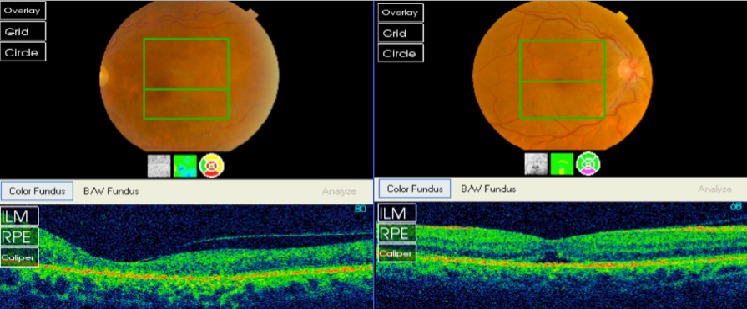
Amélioration du fond d’œil et disparition du liquide sous rétinien à l'OCT après 8 mois d’évolution

Nous rapportons le cas d'un patient de 54 ans suivi pour myélome multiple IGA Kappa stade III A depuis 2 mois, qui se présente pour une BAV bilatérale depuis 6 mois. L'examen ophtalmologique trouve une acuité visuelle à 6/10 au niveau de l’œil droit et à 2 mètres au niveau de l’œil gauche. L'examen du segment antérieur est sans particularité en ODG. Le tonus oculaire est normal aux deux yeux. L'examen du fond d’œil révèle une rétinopathie de stase liée à l'hyperviscosité sans OVCR véritable ou « Paraproteinaemicus fundus » de l’œil droit et une OVCR au niveau de l’œil gauche (Figure 1). L'angiographie à la fluorescéine montre une OVCR au niveau de l’œil gauche forme ‘démateuse, avec importantes dilatations et tortuosité veineuses et une rétinopathie de stase liée à l'hyperviscosité sans OVCR véritable de l’œil droit (Figure 2). La tomographie par cohérence optique révèle la présence bilatérale du liquide sous rétinien (Figure 3). Le patient est adressé à son médecin traitant pour une éventuelle cure de chimiothérapie. L’évolution est marquée par la disparition du DSR en ODG avec amélioration de l'AV (8/10 en OD et 1/10 en OG) après 8 mois d’évolution (Figure 4).

## Discussion

Les occlusions veineuses rétiniennes représentent une cause importante de perte de la vision, en effet, c'est la rétinopathie d'origine vasculaire la plus fréquente, après la rétinopathie diabétique [3]. Le syndrome d'hyperviscosité est un ensemble de symptômes et de signes cliniques qui reflètent une concentration sérique accrue d'une protéine monoclonale [4]. L'hyperviscosité sanguine est un facteur important dans le déclenchement des OVR. Elle peut être due à [5]: une augmentation des éléments figurés du sang, par exemple dans les hémopathies: polycythémie primaire ou secondaire insuffisance cardiaque ou pulmonaire, une insuffisance rénale, une déshydratation; hyper-éosinophilie; thrombocythémie; leucémie; certains lymphomes; l'augmentation de la fraction protéique du sang comme dans les dysglobulinémies: macroglobulinémie de Waldenstrom essentiellement car les IgM sont les globulines de plus fort poids moléculaire; maladie de Kahler; cryoglobulinémie.

Le syndrome d'hyperviscosité est le plus souvent associé à la maladie de Waldenström, mais ila été rapporté dans d'autres pathologies à savoir le myélome multiple, la maladie de la chaine légère k, la cryoglobulinémie et plus rarement la polyarthrite rhumatoïde [6]. La rétinopathie du syndrome d'hyperviscosité est liée au ralentissement circulatoire qui affecte de manière prépondérante le secteur veineux et donne un aspect de rétinopathie de stase bilatérale, avec dilatation et tortuosité de l'ensemble des veines rétiniennes. A un certain degré d'hyperviscosité, une occlusion veineuse véritable peut survenir [5]. Le diagnostic est basé sur l'examen physique et le bilan biologique, un traitement rapide conduit à une reprise de la vision. Les troubles visuels sont fréquents chez les patients atteints du syndrome d'hyperviscosité avec un aspect pathognomonique du fond d’œil: « paraproteinaemicus fundus ».

Dans le paraproteinaemicus fundus, on note une tortuosité et un engorgement des veines rétiniennes qui prennent un aspect « saucisse like ». En l'absence du traitement, cette entité peut alors progresser à une véritable occlusion de la veine centrale de la rétine [6]. En cas d'OVCR secondaires à une macroglobulinémie de Waldenstrom, les plasmaphérèses ont un effet spectaculaire sur l'aspect du fond d’œil [7]. Pour les OVCR compliquant un myélome multiple, le soutien clinique et la chimiothérapie parviennent à contrôler effectivement la production des paraprotéines et à amélioration les symptômes, même avoir recours sans la plasmaphérèse [2]. Les caractéristiques du fond d’œil peuvent servir comme des paramètres d’évaluation du traitement [8]. Ces occlusions symptomatiques surviennent généralement chez des sujets plus jeunes et leur évolution est fonction du pronostic de la maladie générale [5].

## Conclusion

Les occlusions veineuses rétiniennes (OVR) secondaires aux syndromes d'hyperviscosité sont rares. Le syndrome d'hyperviscosité doit être recherché chez tout patient ayant un cancer qui se présente avec la triade: saignement, symptômes visuels, et manifestations neurologiques [9]. Le diagnostic peut être difficile en raison de symptômes non spécifiques tels que la fatigue, anorexie, faiblesse, et des troubles visuels. Le traitement comprend la réhydratation, phlébotomie et plasmaphérèse [10].
